# Tailored Therapies in Addiction Medicine: Redefining Opioid Use Disorder Treatment with Precision Medicine

**DOI:** 10.3390/jpm15080328

**Published:** 2025-07-24

**Authors:** Poorvanshi Alag, Sandra Szafoni, Michael Xincheng Ji, Agata Aleksandra Macionga, Saad Nazir, Gniewko Więckiewicz

**Affiliations:** 1 Department of Psychiatry, Texas Tech University Health Sciences Center, Lubbock, TX 79430, USA; poorvanshi.alag@ttuhsc.edu (P.A.); michael.ji@ttuhsc.edu (M.X.J.); saad.nazir@ttuhsc.edu (S.N.); 2 Department of Psychiatry, Faculty of Medical Sciences in Zabrze, Medical University of Silesia, 41-808 Zabrze, Poland; sandra.a.szafoni@gmail.com (S.S.);

**Keywords:** opioid use disorder, precision medicine, individualized treatment, pharmacological therapies, methadone, buprenorphine, naltrexone, long-acting medications, addiction psychiatry

## Abstract

Opioid use disorder (OUD) is a chronic disease that remains difficult to treat, even with significant improvements in available medications. While current treatments work well for some, they often do not account for the unique needs of individual patients, leading to less-than-ideal results. Precision medicine offers a new path forward by tailoring treatments to fit each person’s genetic, psychological, and social needs. This review takes a close look at medications for OUD, including methadone, buprenorphine, and naltrexone, as well as long-acting options that may improve adherence and convenience. Beyond medications, the review highlights the importance of addressing mental health co-morbidities, trauma histories, and social factors like housing or support systems to create personalized care plans. The review also explores how emerging technologies, including artificial intelligence and digital health tools, can enhance how care is delivered. By identifying research gaps and challenges in implementing precision medicine into practice, this review emphasizes the potential to transform OUD treatment. A more individualized approach could improve outcomes, reduce relapse, and establish a new standard of care focused on recovery and patient well-being.

## 1. Introduction

Substance use disorders (SUDs) pose a critical challenge not only to modern psychiatry but across the entire healthcare system. These disorders include dependence on alcohol, tobacco, and illicit drugs—contributing to substantial health complications, social instability, and economic strain at both individual and societal levels [1]. According to the World Health Organization (WHO), in 2019, excessive alcohol use was responsible for approximately 2.6 million deaths globally, accounting for 4.7% of all global deaths [2]. The use of psychoactive substances, including illicit drugs, contributed to an estimated 600,000 deaths annually [2]. Beyond mortality, SUDs are associated with chronic physical and mental health conditions, greater healthcare demands, and significantly burden public health systems worldwide [1].

Although alcohol and tobacco remain the most widely used substances, the surge in opioid use in the past two decades has created a public health emergency [3]. Opioid use disorder (OUD) is a chronic, relapsing condition characterized by the compulsive use of opioids despite harmful consequences. It involves both physical dependence and psychological cravings, often leading to significant impairment in health, social functioning, and quality of life. The opioid crisis, particularly in North America, has been escalated by intensified by the widespread prescription of opioid analgesics and the increased availability of illicit synthetic opioids, particularly fentanyl [4]. In 2022, the United States recorded an estimated 111,029 drug overdose deaths, with 84,181 (75.8%) attributed to opioids, primarily fentanyl and other synthetic opioids, excluding methadone [5]. Preliminary data for 2023 show there is a slight decline; the total number of overdose deaths decreased to 107,543, while opioid-related fatalities declined to 81,083, accounting for 75.4% of all overdose deaths [5]. It is important to note that the data for 2023 are provisional and may be subject to revision as additional information becomes available.

In Europe, data may underreport the situation due to incomplete national data and limited toxicological infrastructure. The trend is similarly concerning. In 2020, more than 5800 drug-induced deaths were recorded in the European Union (EU)—a 12% increase from the previous year—where opioids were detected in nearly three-quarters of cases [6]. Preliminary estimates for 2022 indicate approximately 6400 drug-induced deaths, with opioids remaining the primary agents [6].

These mortality trends emphasize the urgent need for effective and accessible treatment strategies for opioid use disorder. The most evidence-based approach involves medications for opioid use disorders (MOUDs), which include methadone, buprenorphine, and naltrexone [7]. These medications alleviate withdrawal symptoms and reduce cravings, supporting sustained recovery. Substantial evidence suggests that these interventions significantly reduce rates of illicit opioid use, improve treatment retention, and significantly reduce the risk of overdose [8,9,10]. However, despite its proven efficacy, MOUD is underutilized, and treatment outcomes vary widely among individuals [11].

A complex interplay of biological, psychological, and social determinants influences response to treatment. Standardized protocols may fail to adequately serve patients with adequately with co-morbidities. Optimizing care requires more personalized and integrated approaches that incorporate current scientific advancements—including genetics, epigenetics, and the social determinants of health.

Recent advancements in pharmacogenetics and epigenetics have provided critical insights into the variability of opioid metabolism among individuals, the associated risk of addiction, and the differential treatment responses [12]. These insights pave the path for personalized pharmacological therapies that could improve individual outcomes. Additionally, machine learning (ML) and artificial intelligence (AI) technologies offer new tools to predict relapse, monitor adherence, and tailor treatment regimens in real time [13,14,15].

Social components such as housing insecurity, income level, and access to supportive resources exert a powerful influence over treatment adherence and long-term success [16,17]. Incorporating these elements is essential for developing more holistic, patient-centered care for opioid use disorder (OUD).

This review synthesizes the most recent research on OUD treatment, with a focus on pharmacological strategies, genetic and technological advances, and the psychosocial determinants that shape treatment success. By integrating these perspectives, we aim to propose a comprehensive and personalized approach to improving outcomes for individuals with OUD. To the best of our knowledge, this is the first review of its kind to integrate new technologies, psychosocial determinants, and practical biomarkers.

## 2. Methodology

This manuscript is a narrative review aimed at synthesizing current knowledge on Opioid Use Disorder (OUD), with a particular focus on pharmacological treatment strategies, precision medicine application, and emerging technological innovations. The review was conducted by a team of psychiatry trainees and subsequently reviewed for accuracy and clinical relevance by two subject matter experts in the field of addiction medicine and mental health. To gather the relevant literature, comprehensive searches were conducted across academic databases: PubMed, Web of Science, Scopus, Embase, and EBSCO databases. Both MeSH terms and free-text keywords were used in search queries. Search terms included combinations of “Opioid-Related Disorders”, “Medication-Assisted Treatment”, “Methadone”, “Buprenorphine”, “Naltrexone”, “Naloxone”, “Precision Medicine”, “Pharmacogenomics”, “Machine Learning”, “Artificial Intelligence”, and “Methylation”, among others. Searches were limited to articles published in English. We did not restrict our literature search to any specific publication year; however, the oldest article included dates back to 2005. A total of 69 articles were included, and their quality was assessed by the project supervisors, who are experts in the field of addiction medicine.

Inclusion criteria focused on peer-reviewed sources such as original research articles, systematic and narrative reviews, meta-analyses, clinical guidelines, and retrospective cohort studies. Manual screening of bibliographies and forward citation tracking were also conducted to identify additional relevant sources. For epidemiological data and health-policy context, authoritative sources were consulted, including reports from the World Health Organization (WHO), the Centers for Disease Control and Prevention (CDC), and the European Monitoring Centre for Drugs and Drug Addiction (EMCDDA).

Topic-specific literature reviews were also performed for sections addressing psychosocial and demographic factors, pharmacogenomics, and digital health technologies. These targeted searches included terms such as “socioeconomic factors”, “access to care”, and “subgroup differences in OUD treatment”, and were filtered based on relevance, publication in peer-reviewed journals, and recency. Where necessary, full-text access to paid articles was provided through institutional resources. The resulting synthesis reflects a broad yet focused examination of the most current and clinically relevant advancements in the treatment and management of opioid use disorder.

## 3. Classical Approach—How Is OUD Treated Right Now?

Research robustly demonstrates that the gold standard for treating opioid use disorder (OUD) is medications for opioid use disorder (MOUD), which includes three FDA-approved medications—namely, buprenorphine, naltrexone, and methadone—in combination with behavioral health interventions [18]. However, in acute cases of an opioid overdose, naloxone, an opioid antagonist, remains the recommended emergency reversal medication [19,20]. The combined approach of MOUD with behavioral interventions has been shown to be significantly more effective than non-medicated regiments. Treatment that excludes pharmacological support is associated with significantly elevated risks of relapse, engagement in high-risk behaviors, such as syringe sharing, and opioid overdose-related mortality [18]. A 2018 study found that individuals receiving only behavioral health services had a median retention time that was 1.8 times shorter than those treated with buprenorphine, 2.2 times shorter than those treated with naltrexone, and 4.8 times shorter than individuals treated with methadone [21].

Furthermore, interventions that focus solely on mitigating withdrawal symptoms—such as α2 agonists, β-blockers, antidiarrheals, and antiemetics—may provide temporary relief but do not address opioid cravings. Consequently, these treatments have been proven markedly less effective than MOUD in achieving sustained recovery [22]. It is crucial to emphasize that opiate withdrawal management alone, without concurrent behavioral health support or MOUD, is neither recommended nor recognized as a standalone treatment [19].

The duration of treatment is vital, as longer continuous treatment is associated with delayed relapse and a lower likelihood of re-entry into treatment re-entry—an indicator commonly used to assess relapse [23]. The primary medications employed in MOUD can be categorized based on their mechanisms of action: methadone as a full μ-opioid agonist, buprenorphine as a partial μ-opioid agonist, and naltrexone as a long-acting opioid antagonist. Whereas naloxone, used for overdose reversal, is a short-acting μ-opioid antagonist [20,22].

Methadone has demonstrated efficacy in OUD treatment; however, its pharmacokinetics pose challenges due to a highly variable elimination half-life ranging from 5 to 130 h. This variability necessitates precise dosing strategies to mitigate opioid-related adverse effects [24]. These include respiratory depression and dose-related risk of torsade de pointes, a form of ventricular tachycardia caused by QT interval prolongation [25]. In the United States, methadone access is limited than other OUD medications, as it can only be ordered from opioid treatment program clinic providers with specialized licensure, limiting its availability in office-based settings [25]. Despite these limitations, both methadone and buprenorphine have been shown to significantly improve overall survival in individuals with OUD [22].

While buprenorphine monotherapy is generally not the preferred treatment modality, it may be prescribed in specific circumstances, such as for individuals who are pregnant, hypersensitive to naloxone, have hepatic impairment, or are undergoing a transition from methadone [26].

Both methadone and buprenorphine are effective in suppressing opiate withdrawal symptoms and mitigating the euphoric effects of other opioids. Consequently, patients treated with these medications will develop physical dependence on them. Additionally, there is also a risk of diversion and misuse [18].

The combination of buprenorphine and naloxone is widely recommended as a first-line treatment due to its lower risk of respiratory depression and fatal overdose compared to monotherapy with a full opioid agonist such as methadone [18]. Mortality rates have been observed to be up to 6% lower among individuals treated with buprenorphine, including buprenorphine–naloxone combinations, compared to those on methadone monotherapy [27]. Moreover, the buprenorphine–naloxone combination offers greater flexibility for take-home dosing, which is an important consideration in clinical settings [18].

In contrast to full and partial opioid agonists, naltrexone poses no risk of treatment. Naltrexone, available in both oral and extended-release injectable formulations, is a μ-opioid receptor antagonist that prevents endogenous and exogenous opioids from binding to μ-opioid receptors. Due to the risk of precipitated withdrawal, naltrexone is most appropriate for patients who have been opioid-free for a minimum of 7 to 10 days [18,20]. Optimal outcomes are best suited in individuals with high motivation, as relapse following naltrexone treatment carries a heightened risk of overdose due to reduced opioid tolerance [20]. Meta-analyses further indicate that injectable or implantable naltrexone formulations are more effective than oral formulations in managing opioid dependence [28]. The summary of classical approach medications has been provided in Table 1.

## 4. Novel Approach

### 4.1. Genetics and Biomarkers

Opioid use disorder is a condition that is known to have a biological basis that may influence the course of the disorder. With advancements in research and precision medicine, it has become increasingly relevant to understand genetic biomarkers associated with OUD. This understanding would help to develop new treatment approaches and enhance prevention efforts. In this section, we examine the current literature on biomarkers that predict susceptibility to OUD.

There are several genetic biomarkers, and neuroimaging changes have been identified in individuals with OUD. Some of the studies suggest genetic factors contribute to 50% of susceptibility to substance use disorders; however, no specific gene can be identified as causative for OUD. Rather, it is possible that multiple genes together contribute to the disorder. Vorspan et al. (2021) outlined neuroimaging characteristics including altered brain cortical thickness, regional gray matter reduction, and modifications of cortico-subcortical network [29]. Peripheral and systemic neurotrophins have also been suggested historically as potential biomarkers for OUD, but their significance is yet to be well established.

Some of the biomarkers implicated in OUD include opioid receptor genes, dopaminergic system genes, Metabolic Enzyme Genes, along with epigenetic processes that include methylation. Tang et al. (2023) studied DNA methylation as part of the entire process that makes individuals susceptible to OUD. It has been suggested that methylation leads to synaptic plasticity that helps in learning and memory, along with possible involvement in patients with OUD [30]. One extensively studied gene is GAD2, which encodes an enzyme involved in GABA synthesis. Results showed that hypermethylation in the promoter region of GAD2 is associated with opioid use disorder. The same process of hypermethylation has also been implicated in the μ-opioid receptor gene (OPRM1). It is suggested that such hypermethylation leads to reduced gene expression, particularly in the case of GAD2. While a causal link was not established between OUD susceptibility when it comes to GAD2 gene but an association was certainly found [31].

Apart from the mechanism of methylation, there are also ongoing studies about dopamine receptors and their association with OUD. There are five dopamine receptors, but dopamine receptor 3 (D3R) has been implicated and studied widely in OUD. A study by Banks and Sprague (2024) focuses on single-nucleotide polymorphisms of D3R [32]. Specifically, two studies point towards rs324029 and rs2654754 variants that increase the risk of developing OUD. Animal studies where D3R is deleted show that the sensitivity to opioids is increased, indicating that D3R could serve as a prognostic marker for OUD risk. The implications of D3R involvement in OUD have led to trials of D3R antagonists and partial agonists as potential treatments for OUD. Research suggests that changes in D3R, such as downregulation or deranged signaling, can influence the levels of dopamine and hence increase the risk of opioid use. The D3R partial agonist or antagonist may increase the basal tone of dopamine, thereby blocking the reinforcing effect of opioids [33,34]. This is a potential for future investigation and treatment options when it comes to OUD.

A meta-analysis carried out by Huang et al. (2022) found that polymorphisms have population-specific associations with OUD. They found that the rs4680 polymorphism led to increased susceptibility to OUD in the Asian population, but this same association was not observed in Caucasians. Some biomarkers are relevant only to a specific population subset. One study was able to look at how specific genotypes could possibly guide methadone dosing [35]. Specifically, ABCB1 SNP 1236C>T with genotypes C/T or C/C (Jewish) and haplotypes AGCTT carrier, AGCGC heterozygote, or non-carrier (Caucasian) were found to have a lower methadone dose requirement when compared to ABCB1 SNP 1236C>T with genotype T/T (Jewish), who required a higher dose of methadone. Similarly, the CYP biomarkers were also analyzed, and it was found that CYP2B6 genotypes in the Jewish population required lower methadone when compared to a different CYP2B6 genotype in Caucasian patients [35,36,37,38]. These findings further highlight the potential role of genetic biomarkers in tailoring OUD treatment for specific subgroups. Genetic and psychosocial factors in OUD are presented in Table 2.

Pharmacogenomics is a newer approach to evaluate individual response to OUD treatment. The above-mentioned research can be adapted based on genetic profiles. Incorporating pharmacogenomic testing into clinical practice may help tailor opioid prescribing and reduce the risk of developing OUD. With so many ongoing studies and new research, we still have a long way to go when it comes to precision medicine; however, it can be said that we are on the right track to find tailor-made treatments for individuals who are suffering from OUD. Genetic variations need to be further studied across different populations to find out more about these treatment options. Genome-wide association studies are the next step that would help us to find novel genetic biomarkers. As our understanding of these markers grows, the integration of such data into clinical practice could significantly aid in both preventing and treating OUD in genetically at-risk individuals.

### 4.2. Tailoring Therapies by Taking into Account Psychosocial Factors

An important part of addressing the opioid epidemic is looking into key risk factors that contribute to the development of the disorder and barriers to achieving successful treatment outcomes. While we now have effective treatments for OUD, it is estimated that only about 20% of the 2.1–2.4 million individuals diagnosed with OUD in the United States receive treatment. Of those, only one-third receive medications for opioid use disorder (MOUD). Retention rate for these treatment episodes is similarly low at 30–50% [39]. Various psychosocial and demographic factors interplay to create individual risk factors for the development of opioid use disorder, experiencing overdose, not receiving care, or discontinuation treatment prematurely. As the rates of opioid use and opioid-related deaths continue to rise, it is imperative that we take these factors into consideration to identify key areas of intervention—both in treatment and prevention.

Socioeconomic factors exert a significant influence on the development and treatment of opioid use disorder in the population. Lower income, unemployment, and lower educational attainment have all been identified as risk factors for the development of opioid use disorder [40]. Additional studies have also found that the presence of these factors correlates with higher rates of opioid overdose and lower rates of treatment retention [40,41,42]. Social determinants of health, including issues with housing, food, clothing, transportation, utilities, education, and literacy, are strong predictors for the development of an OUD. A majority of individuals diagnosed with OUD in the US rely on Medicaid for healthcare coverage, with eligibility most often based on low income rather than disability. A study on the Medicaid population showed that the presence of any social determinant of health vulnerability resulted in 26% higher odds of being diagnosed with OUD than those without [43]. In more community-based studies, areas experiencing major economic downturns have also shown increases in opioid-related deaths. Populations facing high food insecurity, financial strain, or lower levels of education were more likely to misuse prescription opioids [44]. Rural and lower-income communities also have less access to MOUD, the gold standard for treatment of OUD [45]. The socioeconomic impact of OUD is bidirectional: OUD increases the likelihood of negative socioeconomic outcomes (i.e., higher rates of first-time homelessness) while also disproportionately affecting populations already experiencing these vulnerabilities [44]. Homelessness, in particular, remains a significant barrier as it is associated with both decreased access to MOUD treatment centers and lower rates of receiving MOUD when they are admitted to treatment programs −28% vs. 44% in one study [46]. These findings underscore the need for OUD prevention and treatment programs tailored to lower-income communities that focus on addressing modifiable socioeconomic risk factors and barriers to care.

Racial and ethnic disparities have also played a substantial role in shaping the evolving landscape of OUD. While overall rates of OUD have been relatively similar across racial groups, opioid overdose and mortality rates have been increasing disproportionately among racial and ethnic minorities, particularly Black and Indigenous Americans [47]. Additionally, Black patients are half as likely as Caucasian patients to receive follow-up appointments after surviving a nonfatal overdose [48].

Barriers are also pronounced among racial and ethnic minorities for access to care. Black and Hispanic patients are less likely to receive MOUD during residential treatment—even though retention rates are often higher among these groups when they do receive care [49]. In predominantly Black or Hispanic communities, methadone clinics are more commonly concentrated, where treatment is often delivered through a more disciplinary framework compared to the more flexible and autonomy-focused approach seen in buprenorphine programs [48]. Furthermore, disparities in health literacy and mistrust of healthcare systems may contribute to lower treatment retention among Black Americans with OUD [50]. To bridge the treatment gap and reduce opioid-related mortality among racial and ethnic minorities, it is essential to address the unique barriers to care affecting these communities.

Psychiatric comorbidities, including anxiety, depression, PTSD, and other substance use disorders, are highly comorbid among individuals with OUD. These are linked to a higher risk of developing OUD and increased opioid-related mortality rates when untreated [51]. Risk is higher with concurrent use of benzodiazepines, other illicit substances, or a history of suicidality. Studies show treatment success rates for OUD are highest among patients who also achieve recovery from co-occurring psychiatric and substance use disorders [52]. Therefore, screening for and addressing psychiatric comorbidities is essential for both preventing OUD in at-risk individuals and improving treatment outcomes among those already diagnosed.

Other demographic factors may also warrant consideration when optimizing treatment and prevention strategies for OUD among special populations. A notable increase in OUD prevalence has been observed in younger individuals, particularly adolescents and young adults. Among those identified with OUD, many reported access to opioids from an early age (under 13 years), often through prior prescriptions for themselves or family members [52,53].

Therefore, a key component of prevention should include education and efforts to limit opioid diversion, particularly targeted towards adolescents and their parents.

In contrast, older adults, on the other hand, face higher opioid-related mortality, which may be attributed to increased dose-related sensitivity, medical comorbidities such as obstructive sleep apnea, and concurrent use of medications like benzodiazepines [54]. For them, deprescribing practices and addressing medical and psychiatric comorbidities may offer more benefit.

While sex-based differences in OUD risk and treatment response are not consistently observed, women with OUD are more likely to present with co-occurring mental health conditions, psychosocial challenges, and histories of physical or sexual violence. These factors contribute to poorer treatment outcomes and should be appropriately screened for and addressed [55]. Pregnant women are a particularly vulnerable population due to a lack of providers willing to treat them and concerns about legal consequences. Despite this, they stand to benefit as treatment with methadone or buprenorphine for mothers with OUD results in improved maternal and infant outcomes compared to supervised withdrawal or no treatment [56]. Buprenorphine in particular may be preferred for older adults and pregnant women, given its better safety profile. Another at-risk population includes individuals with a history of criminal convictions. This group is at heightened risk of developing OUD, experiencing fatal overdose, and demonstrating lower retention rates in treatment programs [40,57]. Despite this, fewer than half of jails in the United States currently offer treatment for inmates with OUD [58], highlighting a major gap in access and a unique opportunity for targeted intervention. As previously discussed, FDA-approved medications for opioid use disorder (methadone, buprenorphine, naltrexone) have been identified as the gold standard for treatment, given extensive evidence in reducing opioid cravings and withdrawal, reducing illicit opioid use, decreasing risk of overdose, increasing retention in treatment programs, and improving social/occupational functioning [45,59]. Despite this, less than half of the patients who seek treatment for OUD receive these medications [39].

In addition to access limitations, stigma remains one of the most significant barriers to OUD treatment. Common to all substance use disorders, addiction is often mischaracterized as a personal or moral failure as opposed to a medical condition. OUD is unique in that while its agonist medications offer the most effective treatment, they are viewed with greater stigma than other treatments or not getting treated at all [60]. Stigma can be present at the individual, provider, community, and institutional levels. Individuals may not feel comfortable seeking care for OUD, and they or members of their community may have negative misperceptions regarding agonist medications as “replacing one addiction with another.” Similarly, institutions and policymakers may deprioritize or inadequately support the implementation of these treatments. These multilevel barriers compound existing constraints, further reducing access to care. Therefore, patient and community education, provider training, and structured outreach programs are essential components that must be integrated into treatment strategies for OUD.

Currently, there is no established evidence-based primary or secondary prevention program for OUD in adults. As we have seen here, a variety of psychosocial factors contribute to creating individual risks for OUD and overdose, as well as individual barriers to accessing treatment. Predictive analyses, particularly those employing machine learning models, have shown the ability to stratify risk among populations for developing OUD, experiencing an overdose, and prematurely discontinuing treatment [61,62]. Using this, we may be able to identify individuals most at risk for developing OUD to target outreach programs towards. We can also identify modifiable risk factors to address that will reduce the risk of developing the disorder, experiencing relapse, or an overdose. Likewise, we can find individual barriers to care that may prevent individuals from seeking treatment or cause them to discontinue treatment early. By incorporating this information into treatment planning, we may move toward individualized strategies that outperform broadly applied, one-size-fits-all approaches—ultimately reducing opioid-related morbidity and mortality.

### 4.3. New Technology—How Artificial Intelligence, Deep Learning, and Digital Tools May Assist with Incorporating These Factors (Genetic and Psychosocial) into Treatment as Well as Improve Treatment Accessibility/Outcomes

The integration of artificial intelligence (AI), deep learning, and digital tools is revolutionizing precision medicine very widely, and it is also doing so in the treatment of opioid use disorder (OUD). These advancements address genetic predispositions, psychosocial influences, and treatment accessibility to enhance outcomes through deep and fast data analysis. As AI technologies continue to evolve, they provide dynamic strategies that address the multifaceted nature of OUD. AI excels at analyzing vast datasets, including genetic profiles, clinical histories, behavioral patterns, and environmental factors. By integrating these data points, AI models can predict individual responses to various treatment strategies. For instance, machine learning models have been utilized to assess genetic polymorphisms associated with opioid metabolism and response, enabling physicians to select medications with the highest likelihood of success for each patient [63].

AI-powered algorithms have demonstrated remarkable capabilities in analyzing complex genomic data to identify genetic markers associated with OUD vulnerability. These insights enable tailored interventions, such as personalized medication plans and targeted behavioral therapies, improving treatment efficacy [64]. For instance, DeepBiomarker2 employs deep learning and natural language processing to predict the risk of substance use disorder based on electronic health records and psychosocial factors in patients diagnosed with post-traumatic stress disorder [65].

In addition to genetic insights, AI-driven systems effectively incorporate psychosocial factors such as socioeconomic status, stress levels, and environmental triggers into treatment strategies. Machine learning models have shown promise in predicting relapse risks and identifying individuals who require intensified psychosocial support [66]. This approach ensures more comprehensive care by integrating both biological and environmental factors.

Moreover, digital health platforms enhance treatment accessibility through remote monitoring, telemedicine, and mobile applications. AI-driven virtual counseling systems provide real-time interventions, mitigating geographical and logistical barriers to care [67]. Tools like conversational agents powered by natural language processing deliver cognitive behavioral therapy (CBT) and psychosocial support remotely, significantly improving patient engagement and adherence to treatment plans [68].

Furthermore, wearable devices equipped with AI algorithms monitor vital signs, detect stress responses, and predict potential relapse events, allowing healthcare providers to intervene proactively [69]. These innovations empower precision medicine by individualizing care strategies and enhancing overall treatment outcomes, but they also come with new challenges, as the application must be guided by strong ethical standards and appropriate regulatory controls, because the use of AI in opioid use disorder (OUD) treatment raises important ethical and regulatory concerns. Key issues include data privacy, especially given the sensitivity of health and substance use information, and algorithmic bias, which can lead to unequal outcomes across populations if not properly addressed. Transparency and accountability are also critical, as AI tools must provide interpretable outputs to support, not replace, clinical judgment. Regulatory oversight is still evolving, but frameworks from agencies like the FDA emphasize the need for safety, effectiveness, and ongoing evaluation of AI systems in healthcare settings.

## 5. Discussion

While opioid-related mortality is a critical concern, it represents only one aspect of the broader burden of opioid use disorder. Chronic opioid misuse, whether illicit or prescribed, can lead to significant physical, psychological, and social consequences, including tolerance, dependence, cognitive impairment, decreased quality of life, and increased risk of infectious diseases. Additionally, the misuse of prescription opioids contributes to healthcare system strain, reduced workforce productivity, and long-term disability, further emphasizing the need for comprehensive approaches to prevention and treatment.

Despite robust evidence supporting the efficacy of current treatment modalities for opioid use disorder (OUD), particularly MOUD when combined with behavioral interventions—these strategies remain substantially underutilized. FDA-approved medications such as methadone, buprenorphine, and naltrexone consistently demonstrate significant reductions in cravings, relapse rates, and mortality. Behavioral therapies further enhance retention and long-term recovery outcomes. However, only a fraction of individuals with OUD access these interventions. In the United States, fewer than half of individuals diagnosed with OUD receive any form of treatment, and even fewer receive MOUD. A similar concerning pattern is seen in Poland, where systemic barriers limit access to comprehensive care. As opioid-related morbidity and mortality continue to rise, particularly in underserved communities, it is increasingly clear that treatment efficacy alone is not enough—effective implementation is now the central challenge.

Emerging technologies offer transformative potential to bridge this implementation gap. Artificial intelligence (AI), deep learning, and digital health tools can improve the accessibility, quality, and personalization of OUD treatment. Machine learning models are increasingly identifying at-risk individuals through analysis of electronic health records, genetic data, and social determinants of health. These predictive tools can help target preventive outreach to vulnerable populations, such as individuals in economically disadvantaged areas, racial and ethnic minorities, adolescents, and those with co-occurring mental health disorders. AI systems can also stratify relapse risk, allowing clinicians to deliver more targeted and intensive intervention.

Digital platforms such as telemedicine, mobile applications, and virtual counseling can make MOUD and behavioral therapies more scalable and patient-centered. These tools help overcome geographic and logistical barriers, offering particular benefits to rural or low-resource regions in both the U.S. and Poland. In addition, wearable devices and remote monitoring technologies can enhance adherence tracking, detect early relapse signs, and prompt timely clinical responses. Together, these innovations expand not only the reach of treatment but also its quality and responsiveness.

Beyond clinical applications, digital tools also play a vital role in public health education and community engagement. Stigma, misinformation, and lack of awareness continue to deter many individuals from seeking care. AI-powered outreach campaigns, culturally sensitive messaging, and localized interventions can address these barriers and promote greater treatment engagement—especially in communities historically excluded from effective OUD care.

However, integrating these technologies into routine care presents significant structural and systemic challenges. Unequal access to digital infrastructure—including reliable internet, smartphones, or wearables—remains a major obstacle, particularly in low-income or rural settings. Health systems must also navigate regulatory constraints, such as MAT prescribing restrictions, limited reimbursement for digital services, and inconsistent data privacy policies. Additionally, the financial burden of implementing and maintaining these technologies can be prohibitive for already resource-limited institutions. Without coordinated policy reform and sustained investment, the advantages of these tools may remain inequitably distributed, deepening existing disparities in OUD treatment.

To support the implementation of precision medicine in OUD care, researchers should focus on identifying and validating biomarkers and genetic predictors of treatment response. Clinicians can begin integrating individualized data into care decisions and adopt clinical decision support tools as they become available. Policymakers should invest in infrastructure for data sharing, fund research in diverse populations, and establish clear regulations to guide the ethical and equitable use of precision approaches in addiction treatment.

In summary, while existing OUD treatments are effective, their impact is undermined by underutilization and structural barriers. Technology offers promising solutions to enhance access, personalize care, and support vulnerable populations. However, realizing this potential will require overcoming infrastructure, policy, and economic hurdles. Integrating these innovations into mainstream healthcare is not just an opportunity—it is a necessity in the ongoing effort to address the opioid crisis.

## 6. Conclusions

1. Current treatments—especially medications for opioid use disorder (MOUD) combined with behavioral interventions—are effective but remain underutilized, contributing to ongoing increases in opioid use and overdose deaths.

2. Precision medicine offers a promising path forward by tailoring OUD treatment to individuals’ genetic, psychological, and social profiles.

3. Emerging technologies such as artificial intelligence (AI), digital tools, and deep learning can help identify at-risk individuals, optimize treatment strategies, and improve monitoring.

4. Digital health platforms can expand access to MOUD and behavioral therapies, particularly in underserved areas in both the United States and Poland.

5. Major structural barriers remain, including limited access to technology, restrictive policies, high implementation costs, and disparities in healthcare infrastructure.

6. To effectively combat the opioid crisis, healthcare systems must integrate personalized, technology-enabled care while addressing systemic barriers to access and equity.

## Figures and Tables

**Table 1:** Summary of classical approach medications in OUD treatment.

Medication	Mechanism of Action	Formulation	Key Benefits	Limitations
Methadone	Full μ-opioid agonist	Oral	Reduces cravings, effective in long-term retention	QT prolongation risk
Buprenorphine	Partial μ-opioid agonist	Sublingual, tablet, long-acting injection	Lower overdose risk, office-based prescribing	Risk of diversion, concern for precipitated withdrawals.
Buprenorphine + Naloxone	Partial μ-agonist + antagonist	Sublingual film/tablet	Lower misuse potential, take-home flexibility	Risk of precipitated withdrawal
Naltrexone	Opioid antagonist (μ-receptor blocker)	Oral or extended-release injection	No abuse potential, suitable for detoxified patients	Requires being off opioids for 7–10 days, risk of non-adherence

**Table 2:** Genetic and psychosocial factors in OUD.

Biomarker	Implications of OUD
GAD2 (hypermethylation)	Associated with synaptic plasticity; hypermethylation reduces expression
OPRM1 (in μ-opioid receptor gene)	Hypermethylation may inhibit gene expression; linked to OUD
D3R (dopamine receptor 3)	Variants rs324029 and rs2654754 increase OUD risk
COMT rs4680 (Asian populations)	Increased susceptibility in Asians, not in Caucasians
ABCB1 SNP 1236 C>T (methadone dose adjustment)	Genotype affects methadone dose requirement
CYP2B6 (methadone metabolism)	Lower dose requirement in Jewish vs. Caucasian populations

## Data Availability

No new data were created or analyzed in this study. Data sharing is not applicable to this article.

## Abbreviations

The following abbreviations are used in this manuscript:

OUD	Opioid Use Disorder
WHO	World Health Organization
EU	European Union
MOUD	Medications for Opioid Use Disorder
ML	Machine Learning
AI	Artificial Intelligence
CDC	Centers for Disease Control and Prevention
EMCDDA	European Monitoring Centre for Drugs and Drug Addiction
FDA	Food and Drug Administration
CBT	Cognitive Behavioral Therapy
